# Effects of *USF1* SNPs and SNP–Environment Interactions on Serum Lipid Profiles and the Risk of Early-Onset Coronary Artery Disease in the Chinese Population

**DOI:** 10.3389/fcvm.2022.882728

**Published:** 2022-06-15

**Authors:** Peng-Fei Zheng, Lu-Zhu Chen, Hong-Wei Pan, Peng Liu, Zhao-Fen Zheng

**Affiliations:** ^1^Cardiology Department, Hunan Provincial People's Hospital, Changsha, China; ^2^Clinical Research Center for Heart Failure in Hunan Province, Changsha, China; ^3^Institute of Cardiovascular Epidemiology, Hunan Provincial People's Hospital, Changsha, China; ^4^Department of Cardiology, The Central Hospital of ShaoYang, Shaoyang, China

**Keywords:** early-onset coronary artery disease, *USF1*, single-nucleotide polymorphism, lipids, inflammatory factors

## Abstract

**Background:**

Upstream transcription factor 1 (*USF1*) single-nucleotide polymorphisms (SNPs) are significantly associated with serum lipid levels in several different ethnic groups or populations, but their association with lipid levels and the risk of early-onset coronary artery disease (EOCAD) has not been reported in Han populations of southern China.

**Methods:**

Six *USF1* SNPs (rs3737787, rs2774276, rs2516839, rs2516838, rs1556259, and rs2516837) were genotyped by next-generation sequencing (NGS) techniques in 686 control subjects and 728 patients with EOCAD.

**Results:**

The genotypic and allelic frequencies of the *USF1* rs3737787 SNP were significantly different between the control and EOCAD groups. The subgroup analysis identified that the rs3737787T allele was related to a decreased risk of EOCAD, whereas the rs3737787C–rs2774276G–rs2516839A and rs3737787C–rs2774276G–rs2516839G haplotypes were related to an increased risk of EOCAD in men, and the rs3737787C–rs2774276G–rs2516839A and rs3737787T–rs2774276C–rs2516839A haplotypes were correlated with an increased risk of EOCAD in women (*p* < 0.05–0.01). Male rs3737787T allele carriers had lower low-density lipoprotein cholesterol (LDL-C), total cholesterol (TC), and triglyceride (TG) concentrations than the rs3737787T allele non-carriers (*p* < 0.01). The interactions of rs3737787 with alcohol consumption and rs2516839 with smoking affected serum TC and LDL-C levels in men, whereas the interaction of rs3737787 with alcohol consumption affected serum high-density lipoprotein cholesterol (HDL-C) levels and the rs2516839-smoking interaction affected serum TC levels in women (*p*_I_ < 0.001). The expression levels of the *USF1* mRNA, interleukin 1β (IL-1β), tumor necrosis factor-α (TNF-α), and interleukin 6 (IL-6) were significantly lower in controls than in patients with EOCAD, and rs3737787T allele carriers displayed lower IL-1β, TNF-α, IL-6, and *USF1* mRNA expression levels than the rs3737787T allele non-carriers. In addition, IL-1β, TNF-α, and IL-6 expression levels were significantly positively correlated with *USF1* mRNA levels (*p* < 0.01).

**Conclusion:**

Sex-specific correlations were identified between the *USF1* rs3737787T allele with blood lipid levels and the risk of EOCAD. The *USF1* rs3737787T allele affects the risk of EOCAD by modulating serum lipid levels and the expression of inflammatory factors, including IL-1β, TNF-α, and IL-6.

## Introduction

Coronary artery disease (CAD) is the single most important cause of death and disability worldwide. It causes millions of deaths and hundreds of millions of disabilities every year and places a heavy economic burden on the families of patients and society as a whole (1). In the past, CAD mainly affected elderly individuals; however, with changes in lifestyle, the prevalence of CAD in young people has increased rapidly in recent years. CAD occurring in individuals under the age of 50 years is defined as early-onset coronary artery disease (EOCAD) (2). Recent studies have shown that CAD is a complex and multifactorial disorder caused by a combination of factors, including genomic background, changes in blood lipid levels, environmental factors, and unhealthy lifestyles, as well as interactions between these factors (3, 4). Hyperlipidemia is considered one of the most important risk factors for CAD and its complications. As early as 2013, the guidelines on blood cholesterol treatment issued by The American College of Cardiology (ACC)/American Heart Association (AHA) emphasized that lipid-lowering therapy should not only focus on reducing the low-density lipoprotein cholesterol (LDL-C) levels but also recommended that patients with CAD, especially patients suffering from acute coronary syndrome (ACS), should be treated with comprehensive lipid-lowering therapy to further reduce the cardiovascular risk caused by atherosclerosis in adults (5). Furthermore, several recent research findings suggested that comprehensive lipid-lowering therapy, including reducing the levels of LDL-C (6), total cholesterol (TC) (7), and total triglyceride (TG) (7), will further reduce the risk of cardiovascular events compared with simply reducing LDL-C levels (8). Currently, the 6% efficacy of statins is a common problem in clinical practice, and doubling the dose of statins only reduces the plasma LDL-C level by 6.4%. Therefore, the lipid-lowering regimen of a proprotein convertase subtilisin/kexin type 9 (PCSK9) inhibitor combined with statins is recommended for patients with ACS to further reduce the risk of cardiovascular events (9).

Hyperlipidemia is a highly hereditary disease, and accumulating evidence from the studies of families (10, 11) and twins (12) revealed that single-nucleotide polymorphisms (SNPs) account for 10–50% of the variation in blood lipid levels. Familial combined hyperlipidemia (FCHL) represents the most common metabolic and genetic form of dyslipidemia, with an incidence of 1–3% in the general population and an incidence of 20–38% in those with a prior history of myocardial infarction (MI) (13). FCHL is closely related to EOCAD, and up to 10–14% of patients with EOCAD are complicated by FCHL (14). The serum lipid profile of patients with FCHL is characterized by elevated TG and/or TC levels, and some patients also have increased levels of very LDL-C (VLDL-C), apolipoprotein (Apo) B, and LDL-C, and decreased levels of high-density lipoprotein cholesterol (HDL-C) (15).

The pathogenesis of FCHL is very complex. Although the role of environmental factors is very important, the effect of genetic factors on the pathogenesis of FCHL cannot be ignored (16, 17). The upstream transcription factor 1 (*USF1*) gene (also named UEF, FCHL, MLTF, FCHL1, MLTFI, HYPLIP1, and bHLHb11, gene ID: 7391, OMIM: 191523, HGNC: 12593) is located on chromosome 1q23.3 (exon count: 12) and encodes a protein belonging to the basic helix–loop–helix leucine zipper family that functions as a key transcription factor to regulate the expression levels of genes such as FASN, ACC, ACLY, and SREBP-1c related to glucose and lipid metabolism (18, 19). Previously, *USF1* was identified as the first major gene related to FCHL in the Finnish population, and the variation in this gene significantly affects the risk of cardiovascular disease (20). Coon et al. noticed that rs3737787, the SNP that maintained the strongest relation to FCHL, was located on the 3'UTR of *USF1* and correlated significantly with serum LDL-C and TG levels in the Utah population (21). Holzapfel et al. found that the *USF1* rs3737787T allele was significantly related to a reduced risk of type 2 diabetes mellitus (T2DM), and other intron variations, including rs2774276C and rs1556259C alleles, were significantly correlated with reduced LDL-C levels in Caucasian women (22). Reiner et al. indicated that the rs2516837T allele, a genetic variation in the 5'UTR of *USF1*, was significantly correlated with elevated serum LDL-C levels in European Americans (23). The rs2516838 and rs2516839 SNPs are located in the intron and 5'UTR of *USF1*, respectively, and Zeggini et al. found that the rs2516838G and rs2516839C alleles are significantly associated with reduced serum TC levels in the Utah population (24). In addition, Laurila et al. found that the *USF1* rs2516839T and rs1556259T risk alleles are significantly related to increased serum TC and decreased serum HDL-C levels in Australians (25). Based on this evidence, the *USF1* gene and its genetic variants are significantly correlated with serum lipid levels, and this correlation is specific to different ethnic groups. Nevertheless, the association between *USF1* rs3737787, rs2516837, rs2774276, rs1556259, rs2516838, and rs2516839 SNPs and the risk of EOCAD remains unclear and has not been reported in the Han populations of southern China. Therefore, this study was designed to investigate the correlations between six selected SNPs and serum lipid levels and the risk of EOCAD in the Han populations of southern China.

## Materials and Methods

### Sample Size Analysis

The QUANTO program (version 1.2.3) (26) was used to calculate the sample size. The minimum total sample sizes that can achieve statistical efficiency and that can be used to calculate the genotypic frequencies of six SNPs and explore the interaction between SNP–SNP/smoking/alcohol consumption are 506, 1,097, 1,198, and 1,113, respectively.

### EOCAD Group

A total of 728 patients with EOCAD were recruited from the Cardiovascular Department of Hunan Provincial People's Hospital. Their ages ranged from 18 to 49 years, and the average age was 39.25 ± 5.15 years. The diagnosis of EOCAD was based on the findings of electrocardiography, cardiac biomarkers, clinical manifestations, and coronary angiography. Inclusion criteria for patients with EOCAD were as follows: EOCAD was defined as significant coronary artery stenosis (≥50%) in at least one of the three major coronary arteries and/or their major branches (branch diameter ≥ 2 mm) detected by using coronary angiography (27). Subjects with a history of neoplastic, type 1 diabetes (T1DM), thyroid, hematologic, autoimmune, liver, and/or renal diseases were excluded. Study protocols were developed based on the guidelines from the Ethics Committee of Hunan Provincial People's Hospital and the 2008 revision of the Declaration of Helsinki of 1975 (http://www.wma.net/en/30publications/10policies/b3/). All subjects provided written and informed consent.

### Control Group

A total of 686 age- and sex-matched healthy subjects were recruited from the Physical Examination Center of Hunan Provincial People's Hospital. Their ages ranged from 18 to 49 years, and the average age was 39.46 ± 5.18 years. None of these participants suffered from CAD at the time of clinical, biochemical, electrocardiogram, medical history, or imaging examinations, such as 64-slice computed tomography coronary angiography. Meanwhile, subjects with a history of cardiomyopathy, valvular disease, congenital heart disease, other systemic diseases, and/or those who took some medications, including β-adrenergic-blocking agents, hypoglycemic agents, lipid-lowering agents, hormones, or thiazide diuretics, were excluded.

### Blood Sample Collection and Blood Lipid Level Detection

About 12 ml of blood were collected from each subject after fasting for more than 12 h, divided into four equal parts (3 ml for each part), and temporarily stored at −20°C until further analysis. Two parts of the sample were collected and placed in yellow glass tubes for blood biochemical tests or enzyme-linked immunosorbent assays (ELISAs). The third part of the sample was collected in an anticoagulant tube containing ethylenediamine tetraacetic acid (EDTA) for RNA extraction. The fourth part of the sample was collected in tubes containing anticoagulants (14.70 g/L glucose, 4.80 g/L citric acid, and 13.20 g/L trisodium citrate) and was utilized to extract DNA. Serum samples were obtained from blood samples after centrifugation at 3,000 rpm for 10 min, and serum LDL-C, ApoB, ApoA1, HDL-C, TG, and TC levels were measured using an autoanalyzer (Type 7170A; Hitachi Ltd., Tokyo, Japan) at the Clinical Laboratory of Hunan Provincial People's Hospital.

### Epidemiological Analysis

Universally standardized protocols and methods were utilized to perform the epidemiological investigations (28). A standard set of questionnaires was used to collect a detailed past medical history, family history, medication, smoking and alcohol consumption, and demographic characteristics. Information on alcohol included questions about the number of liangs (~50 ml) of rum, corn wine, beer, rice wine, or liquor consumed during the preceding 12 months. Total alcohol consumption for each participant was calculated by summing the contributions of rum, corn wine, beer, rice wine, and liquor. Drinking more than once a month was defined as alcohol consumption while drinking less than once a month was defined as non-alcohol consumption (29). Subjects who had smoked more than 100 cigarettes during their lifetime, even if they were not currently smoking, and who currently smoked more than one cigarette per day were classified as smokers, and other subjects were classified as non-smokers (30). Body mass index (BMI), height, waist circumference, weight, and blood pressure were measured as previously described (31).

### SNP Selection and Genotyping

The *USF1* 6 SNPs were selected based on the following criteria: (1) tagging SNPs were identified using Haploview (Broad Institute of MIT and Harvard, USA, version 4.2), and the latest version of the 1,000 Genome Project Database was utilized to predict the functional SNPs that might be associated with blood lipid parameters. (2) More details on SNPs were obtained from NCBI dbSNP Build 132 (http://www.Ncbi.nlm.nih.gov/SNP/). (3) The minor allele frequency (MAF) of the six selected SNPs was >5%. (4) Six *USF1* SNPs, namely, rs3737787, rs2774276, rs2516839, rs2516838, rs1556259, and rs2516837, were chosen using the block-based method. This step is achieved by marking the degree of linkage imbalance (LD) between SNPs using Haploview (*r*^2^ > 0.8). (5) The six selected SNPs have been reported to be correlated with serum lipid parameters or atherosclerosis in different ethnic groups or populations (21–25, 32). The six SNPs were genotyped using next-generation sequencing (NGS) technology at the Center for Human Genetics Research, Shanghai Genesky Bio-Tech Co. Ltd., China (33). The specific steps for multiplex polymerase chain reaction (PCR) and high-throughput sequencing are described in our previous studies (34).

### Diagnostic Criteria

The normal reference ranges of blood lipid parameters were TG (0.56–1.70 mmol/L), ApoB (0.80–1.05 g/L), ApoA1/ApoB ratio (1.00–2.50), HDL-C (1.16–1.42 mmol/L), ApoA1 (1.20–1.60 g/L), LDL-C (2.70–3.10 mmol/L), and TC (3.10–5.17 mmol/L). Diagnostic criteria for diabetes (35), hypertension (36), normal weight, overweight, and obesity (37) were described in previous studies.

### Definition of Environmental Factors

Sex, age, smoking, alcohol consumption, hypertension, diabetes, and BMI may affect lipid levels. Thus, referring to previous studies (38), we selected these factors as environmental factors to further explore the effect of SNP–environmental factor interactions on serum lipid levels.

### Real-Time Quantitative Reverse Transcription PCR

A total of 90 blood samples from patients with EOCAD and 90 samples from normal controls were randomly selected from the total samples of individuals with different rs3737787 genotypes in both the EOCAD and control groups (30 individuals each with the CC, CT, and TT genotypes in both groups). Total RNA was extracted from isolated whole blood samples (3 ml) according to the instructions provided with the TRIzol kit (Invitrogen, USA). Then, the RNA was reverse transcribed into cDNAs using the PrimeScript RT kit (Takara Bio, Japan). Real-Time Quantitative reverse transcription PCR (qRT–PCR) was performed using cDNAs as templates and glyceraldehyde 3-phosphate dehydrogenase (GAPDH) as an internal reference. qRT–PCR was performed using the ABI Prism 7500 sequence-detection system (Applied Biosystems, USA) and the Taq PCR Master Mix kit (Takara). As shown in Supplementary Figure 1, the products of RT-qPCR had a single melting curve indicating the breakdown of only one PCR. The qRT–PCR data were standardized with the 2^−ΔΔCt^ method. qRT–PCR was performed three times for each sample.

### ELISA

Blood samples (3 ml) from the same batch of subjects detected using qRT–PCR were centrifuged at 3,000 rpm at room temperature for 10 min to collect serum samples. The levels of interleukin 1β (IL-1β), tumor necrosis factor-α (TNF-α), and interleukin 6 (IL-6) in human serum were determined according to the instructions of the ELISA kits (IL-1β, ab46052; TNF-α, ab181421; and IL-6, ab178013). The standard curve of human TNF-α, IL-6, and IL-1β is shown in Supplementary Figure 2.

### Statistical Analyses

SPSS software (Version 22.0) was utilized to perform the statistical analyses. SHEsis software (39) was utilized to calculate the pairwise linkage disequilibrium (LD) and the frequency of haplotypes among the six selected SNPs. The student's unpaired *t*-test was utilized to evaluate the normally distributed quantitative data [means ± standard deviation (SD)]. Since TG levels were not normally distributed, they are presented as median values and quartile ranges, and differences were calculated using the Wilcoxon–Mann–Whitney test. Qualitative parameters, including the numbers of drinkers and smokers, genotype distribution, and sex ratio, were analyzed using the chi-squared test. A standard goodness-of-fit test was utilized to calculate the Hardy–Weinberg equilibrium (HWE). The correlation between genotypes and serum lipid levels was calculated using analysis of covariance (ANCOVA), and a corrected *p*-value was adopted after the Bonferroni correction. Several confounding parameters, including sex, alcohol consumption, diabetes, age, smoking, hypertension, and BMI, were adjusted for the statistical analysis. The 95% confidence intervals (CI) and odds ratios (OR) obtained after adjustment for potential confounders were determined using unconditional logistic regression analysis. The effects of the interactions between the six selected SNPs and alcohol consumption, BMI, and cigarette smoking on serum lipid levels were assessed using factorial regression analysis after controlling for several potential confounders. Pearson's correlation analysis was utilized to determine the interactions between *USF1* mRNA expression and TNF-α, IL-1β, and IL-6 levels.

## Results

### Common and Biochemical Characteristics

No significant differences in age structure, diastolic blood pressure, the proportion of smokers, height, or sex ratio were observed between the control and EOCAD groups. The pulse pressure, weight, systolic blood pressure, glucose, and BMI were significantly higher and the proportion of subjects who consumed alcohol was significantly lower in the EOCAD group than in the control group (Table 1).

**Table 1:** Comparison of demographic, lifestyle characteristics of participants.

**Characteristic**	**Control**	**EOCAD**	***p***
	**(*n* = 686)**	**(*n* = 728)**	
Men/women	335/351	352/376	0.856
Age (years)	39.25 ± 5.15	39.46 ± 5.18	0.446
Height (cm)	162.77 ± 7.78	163.23 ± 7.20	0.253
Weight (kg)	59.94 ± 9.01	63.09 ± 9.72	3.45E-10
BMI (kg/m^2^)	22.61 ± 2.91	23.62 ± 2.95	1.12E-10
Smoking, *n* %	362 (42.6)	357 (45.6)	0.161
Alcohol, *n* %	344 (46.2)	181 (27.0)	<0.001
SBP (mmHg)	129.82 ± 19.55	133.19 ± 22.74	0.0003
DBP (mmHg)	79.19 ± 11.50	78.86 ± 11.62	0.597
PP (mmHg)	50.07 ± 15.10	54.33 ± 17.53	0.000001
Glu (mmol/L)	5.86 ± 1.28	6.20 ± 1.13	2.70E-7

*SBP, Systolic blood pressure; DBP, Diastolic blood pressure; PP, Pulse pressure; BMI, Body mass index; Glu, Glucose; EOCAD, early onset coronary artery disease*.

### Serum Lipid Levels in the Control Group

As shown in Table 2, no significant differences in serum ApoB levels were not observed between the control and EOCAD groups. The serum HDL-C, ApoA1, TC, LDL-C, and TG levels, and the ApoA1/ApoB ratio were significantly lower in the EOCAD group than in the control group.

**Table 2:** Serum lipid levels of participants.

**Lipid parameters**	**Control**	**EOCAD**	***p***	**adjust. *p***
	**(*n* = 686)**	**(*n* = 728)**		
TC (mmol/L)	4.87 ± 0.88	4.48 ± 0.98	2.86E-15	2.53E-8
TG (mmol/L)	1.37 (1.26)	1.29 (0.93)	0.003	0.000001
HDL-C (mmol/L)	1.88 ± 0.45	1.13 ± 0.35	5.20E-195	3.33E-183
LDL-C (mmol/L)	2.83 ± 0.75	2.70 ± 0.97	0.003	0.004
ApoA1 (g/L)	1.43 ± 0.28	1.03 ± 0.37	1.83E-101	2.08E-97
ApoB (g/L)	0.89 ± 0.20	0.89 ± 0.26	0.654	0.632
ApoA1/ApoB	1.69 ± 0.58	1.26 ± 0.62	5.46E-40	1.85E-38

*HDL-C, high-density lipoprotein cholesterol; LDL-C, low-density lipoprotein cholesterol; Apo, Apolipoprotein. TC, Total cholesterol; TG, Triglyceride.*

*p-values were obtained by the Students unpaired t-test or Wilcoxon–Mann–Whitney test.*

*Adjust. p-values were obtained by analysis of covariance (ANCOVA) after adjusting for height, weight, BMI, blood pressure, glucose, age, sex, and smoking and alcohol consumption*.

### Genotypic and Allelic Frequencies

As depicted in Table 3, the genotype distribution of six selected SNPs was consistent with HWE in both the control and EOCAD groups (*p* > 0.05). The genotypic (CC, 50.7%; CT, 39.4%; TT, 9.9% vs. CC, 63.5%; CT, 31.9%; TT, 4.6%; *p* = 3.17E-10) and allelic (C, 70%; T, 30% vs. C, 80.1%; and T, 19.9%; *p* = 9.69E-11) frequencies of the rs3737787 SNP were significantly different between the control and EOCAD groups. Significant differences in the genotypic and allelic frequencies of the other five SNPs, rs2774276, rs2516839, rs2516838, rs1556259, and rs2516837, were not observed between the control and EOCAD groups. In addition, detailed and specific genotype frequency distributions of the six detected SNPs in the control and EOCAD groups are also depicted in Supplementary File 1.

**Table 3:** Genotype and allele frequencies of the six upstream transcription factor 1 (*USF1*) single-nucleotide polymorphisms (SNPs) in control and EOCAD groups [*n* (%)].

**Genotype**	**Control** **686**	**EOCAD** **728**	**Allele**	**Control** **1,372**	**EOCAD** **1,456**
**rs3737787C>T**					
CC	348 (50.7)	462 (63.5)			
CT	270 (39.4)	232 (31.9)	C	966 (70.0)	1,156 (80.1)
TT	68 (9.9)	34 (4.6)	T	406 (30.0)	300 (19.9)
*X2*		43.743			41.883
*P*		3.17E-10			9.69E-11
*P_*HWE*_*	0.15	0.50			
**rs2774276G>C**					
GG	444 (64.7)	468 (64.2)			
GC	210 (30.6)	224 (30.8)	G	1,098 (80.0)	1,160 (79.7)
CC	32 (4.7)	36 (5.0)	C	274 (20.0)	296 (20.3)
*X2*		0.071			0.057
*P*		0.965			0.812
*P_*HWE*_*	0.28	0.17			
**rs2516839G>A**					
GG	296 (43.1)	287 (39.4)			
GA	299 (43.6)	328 (45.1)	G	891 (64.9)	902 (62.0)
AA	91 (13.3)	113 (15.5)	A	481 (35.1)	554 (38.0)
*X2*		2.608			2.724
*P*		0.272			0.099
*P_*HWE*_*	0.280	0.240			
**rs2516838G>C**					
GG	500 (72.9)	538 (73.9)			
GC	166 (24.2)	170 (23.4)	G	1,166 (85.0)	1,246 (85.6)
CC	20 (2.9)	20 (2.7)	C	206 (15.0)	210 (14.4)
*X2*		0.191			0.197
*P*		0.909			0.657
*P_*HWE*_*	0.180	0.170			
**rs1556259A>G**					
AA	165 (24.1)	177 (24.3)			
AG	359 (52.3)	382 (52.5)	A	689 (50.2)	736 (50.5)
GG	162 (23.6)	169 (23.2)	G	683 (49.8)	720 (49.5)
*X2*		0.035			0.031
*P*		0.982			0.860
*P_*HWE*_*	0.25	0.21			
**rs2516837T>C**					
TT	307 (44.8)	332 (45.6)			
TC	300 (43.7)	306 (42.0)	T	914 (66.6)	970 (64.6)
CC	79 (11.5)	90 (12.4)	C	458 (33.4)	486 (35.4)
*X2*		0.506			0.000
*P*		0.776			0.999
*P_*HWE*_*	0.67	0.13			

*EOCAD, early-onset coronary artery disease*.

### Genotypes and the Risk of Diseases

As presented in Table 4, after the Bonferroni correction, only the rs3737787 SNP was correlated with the risk of EOCAD (*p* < 0.008, where a value of 0.05 after adjustment for six variables was considered statistically significant). The codominant model (TT vs. CC, OR = 0.36, 95% CI = 0.23–0.56, *p* = 9.62E-6), dominant model (CT/TT vs. CC, OR = 0.59, 95% CI = 0.48–0.73, *p* = 1.28E-6), recessive model (TT vs. CC/CT, OR = 0.42, 95% CI = 0.27–0.66, *p* = 2.11E-6), overdominant model (TT vs. CC/CT, OR = 0.72, 95% CI = 0.57–0.91, *p* = 0.0022), and log-additive model (T vs. C, OR = 0.62, 95% CI = 0.52–0.74, *p* = 7.62E-5) were considered statistically significant. In addition, a subgroup analysis based on sex revealed that male rs3737787T allele carriers maintained a decreased risk of EOCAD compared with rs3737787T allele non-carriers (CT/TT vs. CC, OR = 0.42, 95% CI = 0.31–0.58, *p*_I_ = 0.0055); however, this phenomenon was not observed in women (Table 5).

**Table 4:** Genotypes of the six *USF1* SNPs and the risk of EOCAD.

**SNP/Model**	**Ref.** **genotype**	**Effect genotype**	**EOCAD** **(OR 95% CI)**	***p***
**rs3737787C>T**				
Codominant	CC	CT	0.64 (0.51–0.81)	1.72E-4
		TT	0.36 (0.23–0.56)	9.62E-6
Dominant	CC	CT + TT	0.59 (0.48–0.73)	1.28E-6
Recessive	CC/CT	TT	0.42 (0.27–0.66)	2.11E-6
Overdominant	CC/TT	CT	0.72 (0.57–0.91)	0.0022
Log-Additive			0.62 (0.52–0.74)	7.62E-5
**rs2774276G>C**				
Codominant	GG	GC	1.03 (0.81–1.31)	0.920
		CC	1.10 (0.66–1.85)	
Dominant	GG	GC + CC	1.04 (0.83–1.31)	0.740
Recessive	GG/GC	CC	1.09 (0.65–1.82)	0.740
Overdominant	GG/CC	GC	1.02 (0.81–1.30)	0.850
Log-Additive			1.04 (0.86–1.25)	0.690
**rs2516839G>A**				
Codominant	GG	GA	1.13 (0.89–1.42)	0.31
		AA	1.28 (0.92–1.78)	
Dominant	GG	GA + AA	1.16 (0.93–1.44)	0.190
Recessive	GG/GA	AA	1.19 (0.88–1.62)	0.250
Overdominant	GG/AA	GA	1.05 (0.85–1.30)	0.640
Log-Additive			1.13 (0.97–1.32)	0.130
**rs2516838G>C**				
Codominant	GG	GC	1.21 (0.93–1.58)	0.330
		CC	1.18 (0.61–2.28)	
Dominant	GG	GC + CC	1.21 (0.94–1.56)	0.140
Recessive	GG/GC	CC	1.12 (0.58–2.15)	0.740
Overdominant	GG/CC	GC	1.20 (0.93–1.56)	0.160
Log-Additive			1.16 (0.94–1.44)	0.170
**rs1556259A>G**				
Codominant	AA	AG	1.00 (0.77–1.30)	0.990
		GG	0.99 (0.73–1.34)	
Dominant	AA	AG + GG	1.00 (0.78–1.27)	0.980
Recessive	AA/AG	GG	0.98 (0.77–1.26)	0.910
Overdominant	AA/GG	AG	1.01 (0.82–1.24)	0.940
Log-Additive			0.99 (0.85–1.16)	0.930
**rs2516837T>C**				
Codominant	TT	TC	1.13 (0.89–1.43)	0.370
		CC	1.26 (0.88–1.81)	
Dominant	TT	TC + CC	1.15 (0.92–1.45)	0.210
Recessive	TT/TC	CC	1.19 (0.85–1.67)	0.310
Overdominant	TT/CC	TC	1.07 (0.85–1.34)	0.570
Log-Additive			1.12 (0.95–1.32)	0.160

*USF1, upstream transcription factor 1; SNP, single-nucleotide polymorphism; EOCAD, early-onset coronary artery disease.*

*p < 0.008 for 0.05 adjusted for six variables was considered to be statistically significant*.

**Table 5:** Associations between the dominant model of rs3737787 and the risk of EOCAD in men and women populations.

**SNP/Genotype**	**Control**	**EOCAD**	**OR [95% CI]**	***p*_I_**
**rs3737787C>T**				
**Men**				
CC	161	236	1	
CT/TT	174	116	0.42 (0.31–0.58)	
**Women**				
CC	187	226	1	
CT/TT	164	160	0.79 (0.58–1.08)	*p*_I_ = 0.0055

*EOCAD, early-onset coronary artery disease; p_I_, the interaction p-value*.

### Relationship Between Genotypes and Serum Lipid Parameters

**Figure 1:**
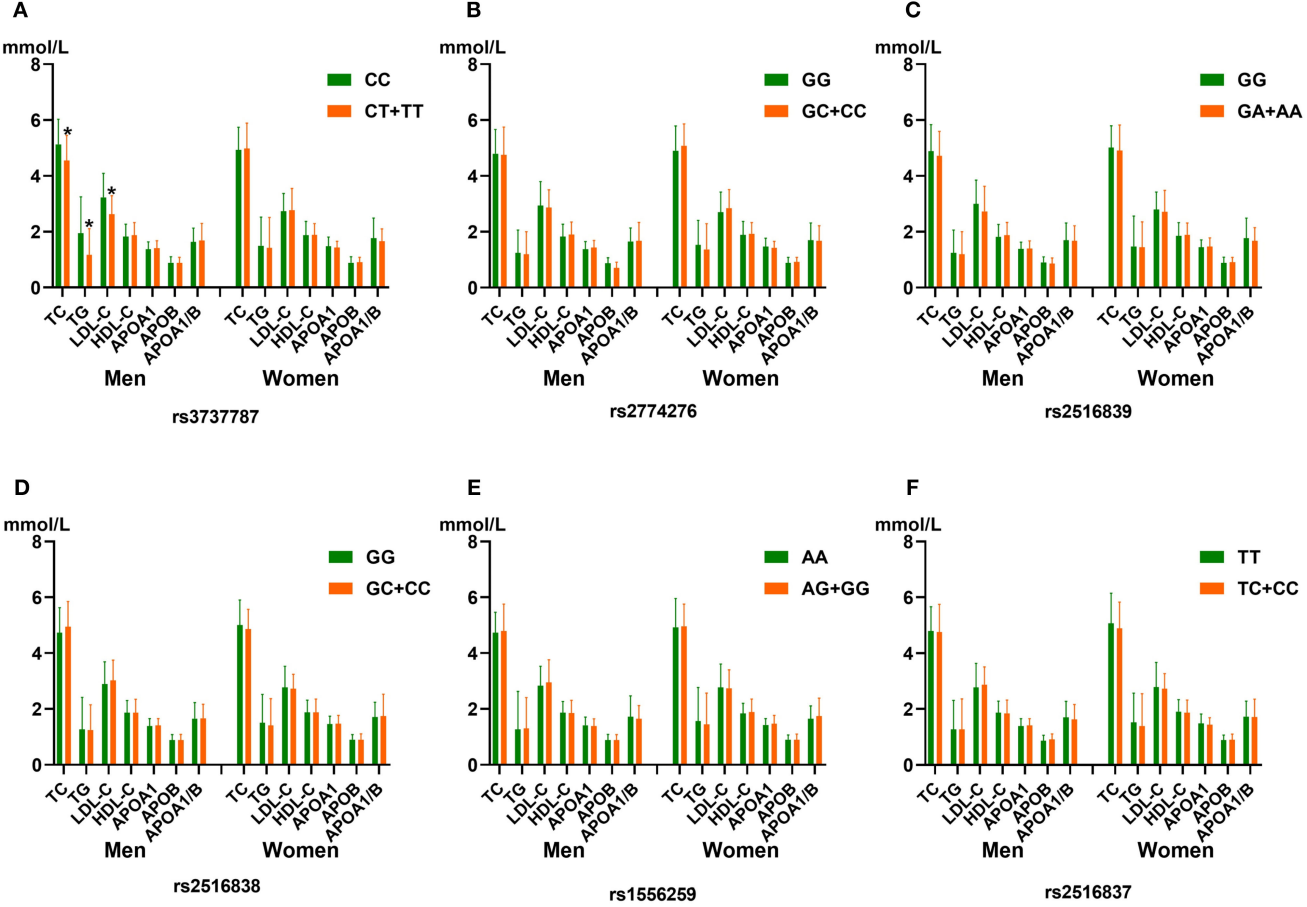
The association between the genotypes of six upstream transcription factor 1 (*USF1*) single-nucleotide polymorphisms (SNPs) and blood lipid levels in men and women from the control group. **(A)** rs3737787C>T, **(B)** rs2774276G>C, **(C)** rs2516839G>A, **(D)** rs2516838G>C, **(E)** rs1556259A>G, and **(F)** rs2516837T>C SNPs. HDL-C, high-density lipoprotein cholesterol; TC, total cholesterol; ApoA1, apolipoprotein A1; TG, triglyceride; ApoB, apolipoprotein B; LDL-C, low-density lipoprotein cholesterol; ApoA1/ApoB, the ratio of apolipoprotein A1 to apolipoprotein B. *p < 0.008 (after adjusting for six independent tests using the Bonferroni correction) was considered to be statistically significant.

Figure 1 shows that rs3737787T allele carriers maintained lower levels of TC, TG, and LDL-C levels than non-carriers in men but not in women (*p* < 0.008, corresponding to *p* < 0.05 after adjusting for six independent tests using the Bonferroni correction). In addition, no significant correlation was observed between the other five SNPs and serum lipid levels (*p* ≥ 0.008 for all).

### Haplotype Frequencies and the Risk of EOCAD

**Figure 2:**
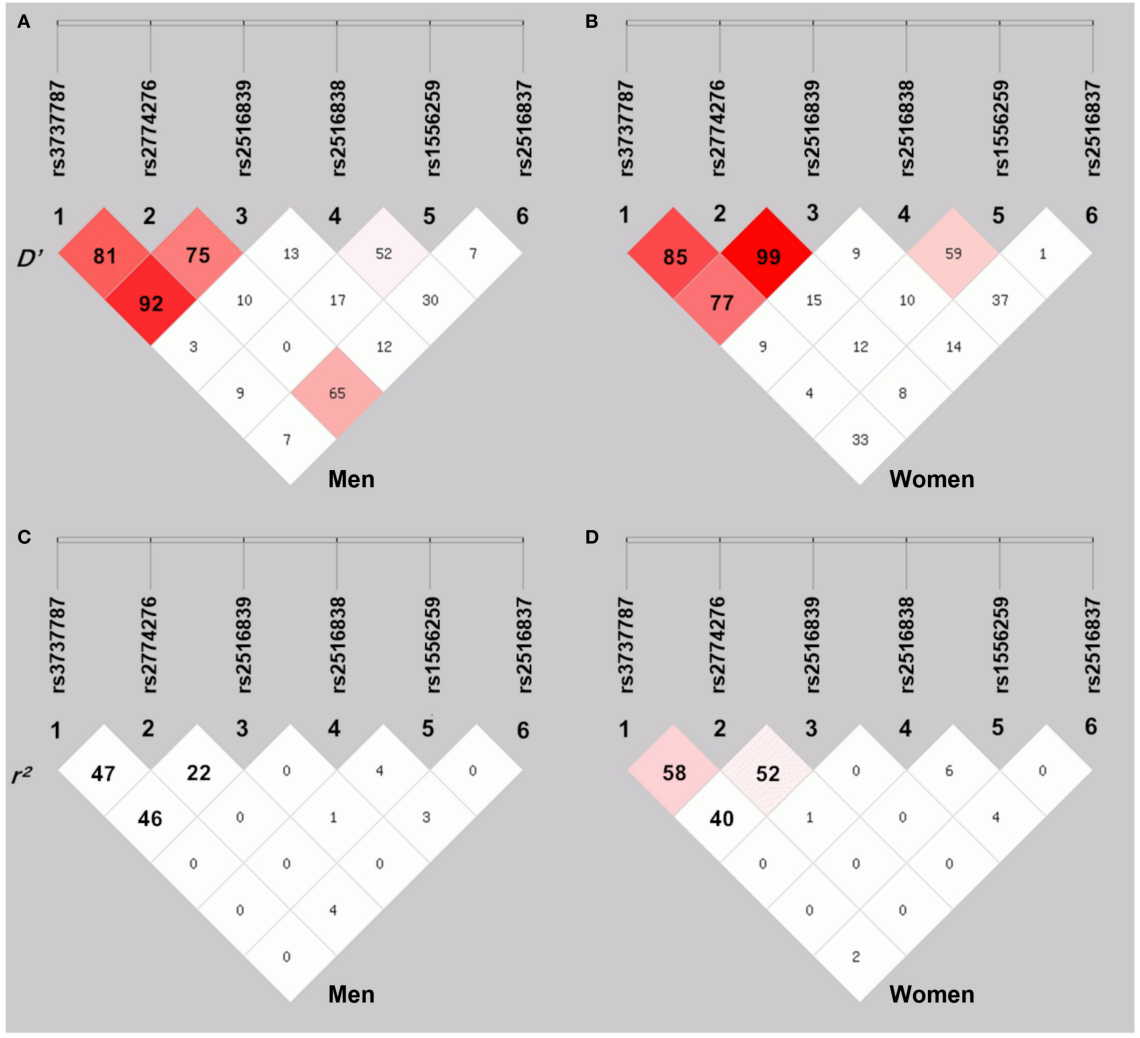
The linkage disequilibrium (LD) represents pairwise *D*' × 100 **(A,B)** and *r*^2^ × 100 **(C,D)** in men and women.

As shown in Figure 2, moderate LD was noted among the rs3737787, rs2774276, and rs2516839 SNPs in men (Figures 2A,C) and women (Figures 2B,D) (*D*', 0.75–0.99 and *r*^2^, 0.22–0.58). Haplotype analyses were performed among the aforementioned three SNPs. As shown in Table 6, the major haplotype is rs3737787C–rs2774276G–rs2516839G. The rs3737787C–rs2774276G–rs2516839A (adjusted OR = 1.967, 95% CI = 1.422–2.720, *p* = 3.52E-005) and rs3737787C–rs2774276G–rs2516839G (adjusted OR = 1.619, 95% CI = 1.298–2.019, *p* = 1.89E-005) haplotypes were correlated with an increased risk of EOCAD in men, and the rs3737787C–rs2774276G–rs2516839A (adjusted OR = 2.951, 95% CI = 1.996–4.362, *p* = 1.86E-008) and rs3737787T–rs2774276C–rs2516839A (adjusted OR = 1.515, 95% CI = 1.158–1.983, *p* = 0.002374) haplotypes correlated with an increased risk of EOCAD in women.

**Table 6:** Haplotype frequencies and the associations between haplotypes and the risk of EOCAD in men and women populations.

**No**.	**Haplotypes**	**Men**				**Women**			
		**Control**	**EOCAD**	**OR [95% CI]**	***p***	**Control**	**EOCAD**	**OR [95% CI]**	***p***
S1	C-G-A	64.75 (0.097)	119.01 (0.169)	1.967 [1.422 ~ 2.720]	3.52E-005	36.74 (0.052)	105.38 (0.140)	2.951 [1.996 ~ 4.362]	1.86E-008
S2	C-G-G	348.60 (0.520)	434.37 (0.617)	1.619 [1.298 ~ 2.019]	1.89E-005	440.95 (0.628)	476.62 (0.634)	1.025 [0.828 ~ 1.268]	0.822770
S3	T-C-A	91.35 (0.136)	113.37 (0.161)	1.255 [0.930 ~ 1.694]	0.136228	107.69 (0.153)	162.00 (0.215)	1.515 [1.158 ~ 1.983]	0.002374

*EOCAD, early onset coronary artery disease.*

*The haplotypes consist of three alleles in the order of rs3737787C>T, rs2274276G>C, and rs2516839G>A SNPs*.

### Effects of SNP–Smoking/Alcohol Consumption/BMI Interactions on Serum Lipid Levels

**Figure 3:**
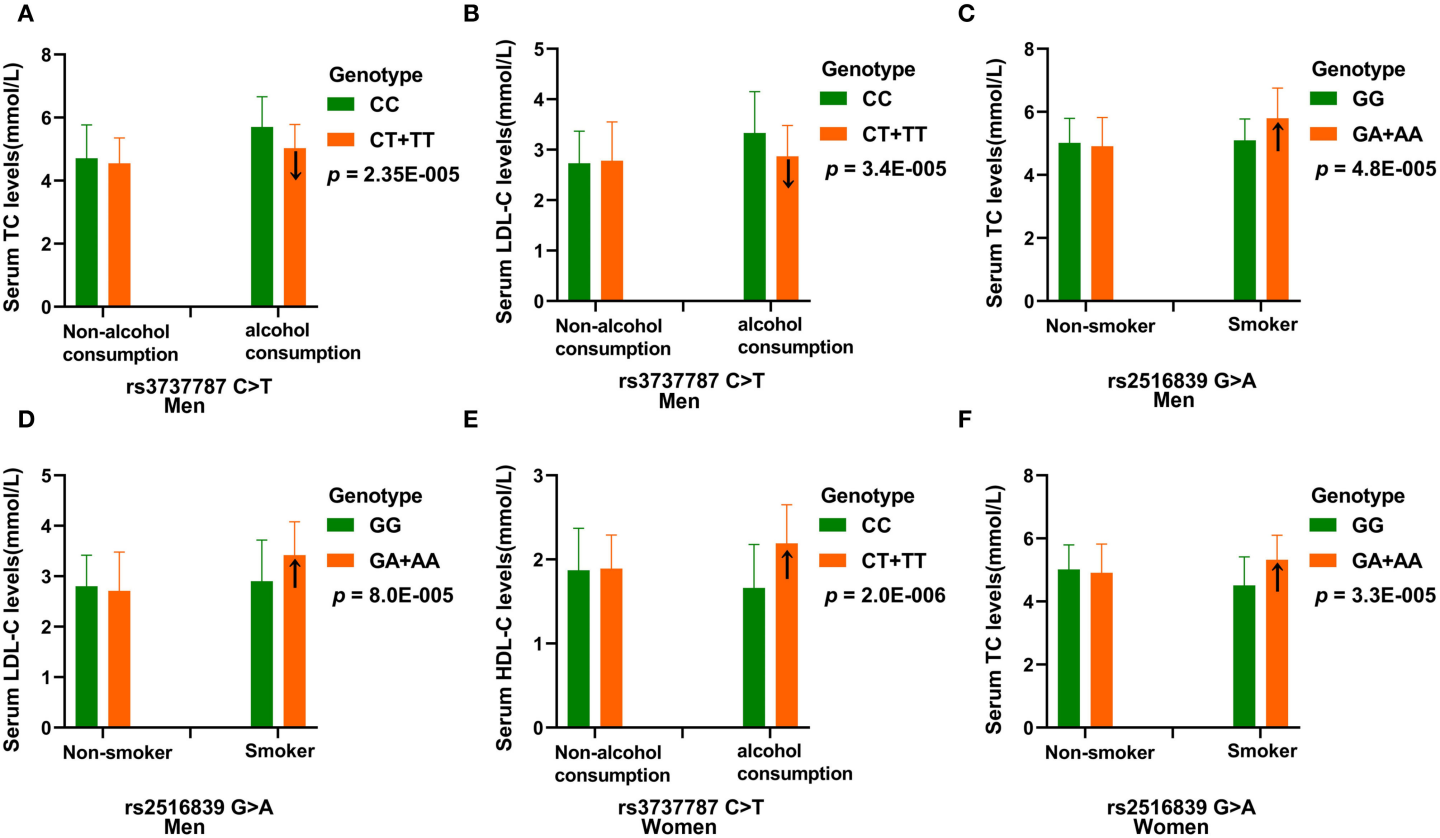
The effect of rs3737787-alcohol consumption and rs2516839-smoking interactions on serum lipid levels. **(A)** Effect of the rs3737787-alcohol consumption interaction on TC levels in men. **(B)** Effect of the rs3737787-alcohol consumption interaction on LDL-C levels in men. **(C)** Effect of the rs2516839-smoking interaction on TC levels in men. **(D)** Effect of the rs2516839-smoking interaction on LDL-C levels in men. **(E)** Effect of the rs3737787-alcohol consumption interaction on HDL-C levels in women. **(F)** Effect of the rs2516839-smoking interaction on TC levels in women. TC, total cholesterol; LDL-C, low-density lipoprotein cholesterol, HDL-C, high-density lipoprotein cholesterol. *p*_I_ < 0.003 was considered statistically significant after the Bonferroni correction (five SNPs × three risk factors).

Table 7 depicts the *p-*values for the interactions (*p*_I_) of SNP–alcohol consumption/smoking/BMI on blood lipid parameters in the control group. The rs3737787 and rs2516839 SNPs interacted with smoking or alcohol consumption to affect serum LDL-C and TC levels in men (*p*_I_ < 0.001, respectively; *p*_I_ < 0.003 was considered statistically significant after the Bonferroni correction: six SNPs × three risk factors). The rs3737787 SNP interacted with alcohol consumption to affect the levels of HDL-C, and the rs2516839 SNP interacted with smoking to affect the levels of TC in women (*p*_I_ < 0.001). As shown in Figure 3, the interaction between rs3737787 CT/TT and alcohol consumption decreased TC and LDL-C levels and smoking increased TC and LDL-C levels in men. In addition, the interaction between rs3737787 CT/TT and alcohol consumption increased HDL-C levels; the interaction between rs2516839 GA/AA and smoking increased TC levels in women.

**Table 7:** The *p*_I_ values for the interactions of genotypes and alcohol consumption, smoking, and BMI on serum lipid levels.

**SNP/Factor**	**Lipid**						
	**TC**	**TG**	**LDL-C**	**HDL-C**	**ApoA1**	**ApoB**	**ApoA1/ApoB**
**Men**							
**rs3737787C>T**							
Smoking	0.030	0.011	0.041	0.046	0.020	0.078	0.351
Alcohol consumption	2.35E-005	0.025	3.4E-005	0.009	0.301	0.115	0.342
BMI	0.401	0.969	0.679	0.385	0.064	0.552	0.498
**rs2774276G>C**							
Smoking	0.145	0.320	0.088	0.029	0.063	0.007	0.394
Alcohol consumption	0.251	0.035	0.143	0.024	0.023	0.106	0.271
BMI	0.445	0.057	0.624	0.537	0.459	0.276	0.226
**rs2516839G>A**							
Smoking	4.8E-005	0.205	8.0E-005	0.080	0.026	0.496	0.088
Alcohol consumption	0.173	0.012	0.011	0.040	0.014	0.510	0.051
BMI	0.021	0.989	0.729	0.237	0.007	0.277	0.397
**Women**							
**rs3737787G>C**							
Smoking	0.473	0.161	0.042	0.775	0.718	0.045	0.220
Alcohol consumption	0.042	0.045	0.293	2.0E-006	0.295	0.104	0.257
BMI	0.270	0.706	0.416	0.249	0.983	0.123	0.023
**rs2774276A>G**							
Smoking	0.013	0.372	0.089	0.712	0.446	0.221	0.037
Alcohol consumption	0.359	0.031	0.142	0.505	0.450	0.026	0.119
BMI	0.950	0.677	0.912	0.462	0.993	0.251	0.073
**rs2516839T>C**							
Smoking	3.3E-005	0.012	0.115	0.654	0.101	0.061	0.321
Alcohol consumption	0.296	0.115	0.235	0.399	0.017	0.055	0.264
BMI	0.217	0.724	0.230	0.300	0.064	0.344	0.886

*SNP, single-nucleotide polymorphism; TC, total cholesterol; TG, triglyceride; HDL-C, high-density lipoprotein cholesterol; LDL-C, low-density lipoprotein cholesterol; ApoA1, apolipoprotein A1; ApoB, apolipoprotein B; BMI, body mass index.*

*p_I_ ≤ 0.003 was considered to be statistically significant after Bonferroni correction*.

### Effect of SNP–SNP Interactions on the Risk of EOCAD

As presented in Table 8, subjects with the rs3737787 TT and rs2516839 GA/AA genotypes; the rs3737787 CT/CC and rs2516839 GG genotypes; the rs3737787 CT/CC and rs2516839 GA/AA genotypes maintained a higher risk of EOCAD than those with the rs3737787 TT and rs2516839 GG genotypes in men (adjusted OR = 1.76, 95% CI = 1.13–2.75, *p* = 0.012; adjusted OR = 2.04, 95% CI = 1.41–2.95, *p* = 1.71E-4; adjusted OR = 2.74, 95% CI = 1.89–3.98, *p* = 1.13E-6) and women (adjusted OR = 1.85, 95% CI = 1.16–2.95, *p* = 0.009; adjusted OR = 2.35, 95% CI = 1.52–3.62, *p* = 1.21E-4; adjusted OR = 3.16, 95% CI = 1.92–5.19, *p* = 5.55E-6).

**Table 8:** Interactions of the rs3737787 and rs2516839 SNPs on the risk of EOCAD.

**SNP/Genotype**	**SNP/Genotype**	**OR [95% CI]**	***p***
**Men**			
rs3737787C>T	rs2516839G>A		
TT	GG	1.00	
TT	GA + AA	1.76 (1.13–2.75)	0.012
CT + CC	GG	2.04 (1.41–2.95)	1.71E-4
CT + CC	GA + AA	2.74 (1.89–3.98)	1.13E-6
**Women**			
rs3737787C>T	rs2516839G>A		
TT	GG	1.00	
TT	GA + AA	1.85 (1.16–2.95)	0.009
CT + CC	GG	2.35 (1.52–3.62)	1.21E-4
CT+CC	GA + AA	3.16 (1.92–5.19)	5.55E-6

*OR, odds ratio; CI, confidence interval*.

### Effects of SNP–Smoking/Alcohol Consumption Interactions on the Risk of EOCAD

As presented in Table 9, the rs3737787 CT/TT–alcohol consumption interaction decreased the risk of EOCAD in both men (adjusted OR = 0.47, 95% CI = 0.29–0.76, *p*_I_ = 0.0024) and women (adjusted OR = 0.70, 95% CI = 0.52–0.93, *p*_I_ = 0.011). The rs2516839 GA/AA–smoking interaction increased the risk of EOCAD in both men (adjusted OR = 2.28, 95% CI = 1.39–3.74, *p*_I_ = 0.00042) and women (adjusted OR = 1.93, 95% CI = 1.14–3.24, *p*_I_ = 0.009).

**Table 9:** Interactions of the genotypes and smoking/alcohol consumption on the risk of EOCAD.

**SNP/Factor**	**Genotype**	**OR [95% CI]**	***p*_I_**	**SNP/Factor**	**Genotype**	**OR [95% CI]**	***p*_I_**
**Men**							
rs3737787C>T				rs2516839G>A			
Non-alcohol consumption	CC	1.00		Non-Smoking	GG	1.00	
	CT/TT	1.25 (0.81–1.92)			GA/AA	0.70 (0.45–1.09)	
Alcohol consumption	CC	1.00		Smoking	GG	1.00	
	CT/TT	0.47 (0.29–0.76)	0.0024		GA/AA	2.28 (1.39–3.74)	0.00042
**Women**							
rs3737787C>T				rs2516839G>A			
Non-alcohol consumption	CC	1.00		Non-Smoking	GG	1.00	
	CT/TT	1.24 (0.88–1.74)			GA/AA	1.07 (0.65–1.78)	
Alcohol consumption	CC	1.00		Smoking	GG	1.00	
	CT/TT	0.70 (0.52–0.93)	0.011		GA/AA	1.93 (1.14–3.24)	0.009

*EOCAD, early-onset coronary artery disease; OR, odds ratio; CI, confidence interval; p_I_, the interaction p-value*.

### Effect of Haplotype–Smoking Interactions on the Risk of EOCAD

As presented in Table 10, the interactions between the rs3737787C–rs2774276G–rs2516839A haplotype and smoking increased the risk of EOCAD in men (adjusted OR = 2.91, 95% CI = 2.15–3.94, *p* = 4.56E-12) and women (adjusted OR = 4.01, 95% CI = 2.90–5.54, *p* = 3.89E-17). The rs3737787C–rs2774276G–rs2516839G-smoking interactions (adjusted OR = 2.57, 95% CI = 1.90–3.47, *p* = 2.51E-12) increased the risk of EOCAD in men, and rs3737787T–rs2774276C–rs2516839A-smoking interactions (adjusted OR = 2.79, 95% CI = 2.08–3.74, *p* = 6.22E-12) increased the risk of EOCAD in women. However, the effects of the haplotype-BMI/alcohol consumption interactions on the risk of EOCAD were not detected in the current study.

**Table 10:** Interactions of the haplotypes and smoking on the risk of EOCAD.

**Haplotype**		**OR [95% CI]**	***p***	**Haplotype**		**OR [95% CI]**	***p***
**Men**							
C-G-A				C-G-G			
Non-Smoking	No-Carriers	1.00		Non-Smoking	No-Carriers	1.00	
	Carriers	2.34 (1.63–3.34)	0.000003		Carriers	2.30 (1.65–3.22)	9.92E-7
Smoking	No-Carriers	1.84 (1.40–2.40)	0.000009	Smoking	No-Carriers	1.44 (0.86–2.41)	0.580
	Carriers	2.91 (2.15–3.94)	4.56E-12		Carriers	2.57 (1.90–3.47)	2.51E-12
**Women**							
C-G-A				T-C-A			
Non-Smoking	No-Carriers	1.00		Non-Smoking	No-Carriers	1.00	
	Carriers	1.91 (1.45–2.52)	0.000005		Carriers	2.36 (1.67–3.34)	0.000001
Smoking	No-Carriers	1.02 (0.75–1.38)	0.918	Smoking	No-Carriers	1.20 (0.92–1.56)	0.0015
	Carriers	4.01 (2.90–5.54)	3.89E-17		Carriers	2.79 (2.08–3.74)	6.22E-12

*EOCAD, early-onset coronary artery disease; OR, odds ratio; CI, confidence interval.*

*The haplotypes consist of three alleles in the order of rs3737787C>T, rs2274276G>C, and rs2516839G>A SNPs*.

### *USF1* mRNA Expression Levels

**Figure 4:**
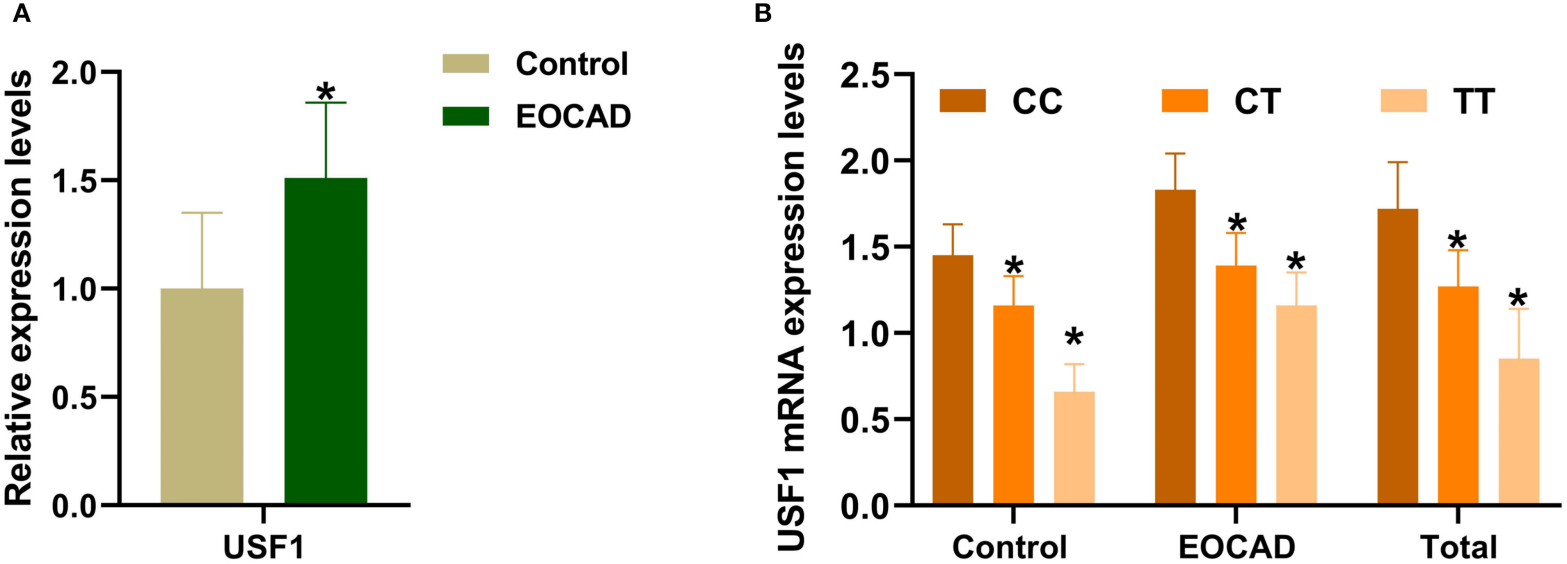
The expression levels of the *USF1* mRNA in the control and EOCAD groups. **(A)** *USF1* expression levels were significantly higher in the EOCAD group than in the control group. **(B)** The rs3737787T allele carriers maintained lower expression levels of the *USF1* mRNA than T non-carriers. *USF1*, upstream transcription factor 1; EOCAD, early-onset coronary artery disease. *p < 0.01.

As shown in Figure 4, the qRT–PCR results revealed a markedly increase in *USF1* mRNA expressionin patients suffering from EOCAD compared with controls (Figure 4A). In addition, we also noticed that carriers of the rs3737787T allele maintained a lower *USF1* mRNA level than rs3737787T non-carriers (Figure 4B).

### TNF-α, IL-1β, and IL-6 Expression Levels

**Figure 5:**
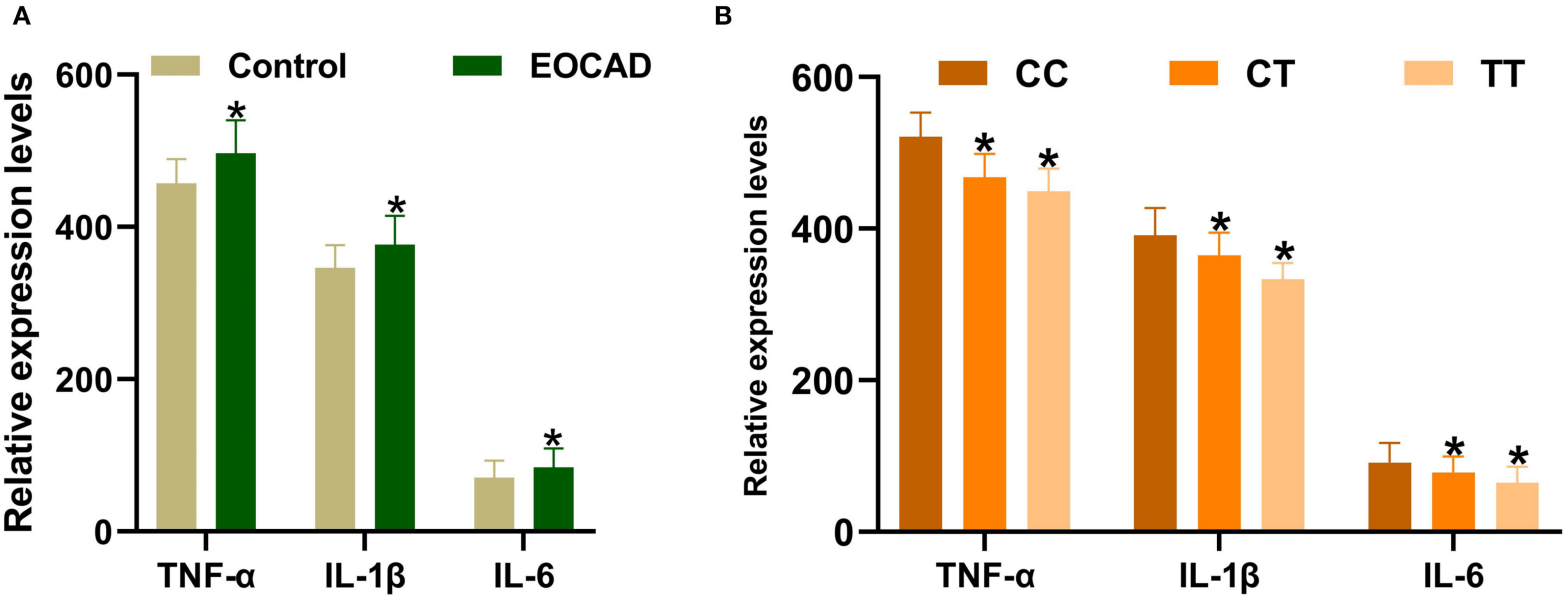
Expression levels of TNF-α, IL-1β, and IL-6 in the control and EOCAD groups. **(A)** Significantly higher expression levels of TNF-α, IL-1β, and IL-6 were detected in the EOCAD group than in the control group. **(B)** The rs3737787T allele carriers maintained lower levels of TNF-α, IL-1β, and IL-6 than T non-carriers. *USF1*, upstream transcription factor 1; EOCAD, early-onset coronary artery disease; TNF-α, tumor necrosis factor-α; IL-1β, interleukin 1β; IL-6, interleukin 6. *p < 0.01.

**Figure 6:**
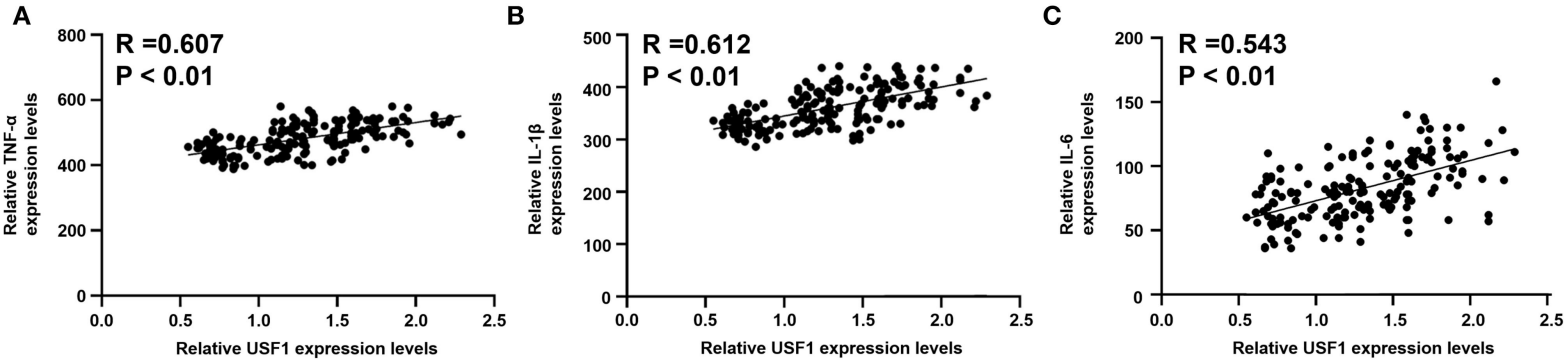
Correlations between *USF1* mRNA expression and serum TNF-α, IL-1β, and IL-6 levels. **(A)** TNF-α levels were positively correlated with the *USF1* mRNA expression levels. **(B)** IL-1β levels were positively correlated with the *USF1* mRNA expression levels. **(C)** IL-6 levels were positively correlated with the *USF1* mRNA expression levels. *USF1*, upstream transcription factor 1; TNF-α, tumor necrosis factor-α; IL-1β, interleukin 1β; IL-6, interleukin 6.

Expression levels of IL-1β, IL-6, and TNF-α were markedly increased in patients with EOCAD compared with control subjects (Figure 5A). Carriers of the rs3737787T allele maintained lower levels of IL-1β, IL-6, and TNF-α than rs3737787T non-carriers (Figure 5B). In addition, Pearson's correlation analysis suggested that the levels of TNF-α (Figure 6A), IL-1β (Figure 6B), and IL-6 (Figure 6C) were positively correlated with *USF1* mRNA expression levels.

## Discussion

As the initial stage of CAD (40), atherosclerosis results from the combination of a chronic inflammatory response (41) and abnormal lipid levels, including reduced levels of Apo A1 (42) and HDL-C (43) along with increased levels of ApoB (42), LDL-C (44), TG (45), and TC (46) in serum. Previous studies have shown that hyperlipidemia, the most common form of dyslipidemia, is essentially a highly heritable disease, and SNPs potentially explain 10–50% of the changes in blood lipid levels (10–12). Meanwhile, a large number of genes and SNPs related to lipid metabolism were also identified as significantly correlated with the risk of CAD (47, 48). The identification of genetic variations related to blood lipid levels and CAD will help to clarify the genetic mechanisms of hyperlipidemia and CAD and will be very important for the prevention and treatment of CAD. Thus, in our current research, we aimed to explore the effects of 6 *USF1* SNPs and their interactions with environmental factors on serum lipid levels and the risk of EOCAD. We noticed that the frequencies of the *USF1* rs3737787 CT and TT genotypes and T allele were significantly lower in patients with EOCAD than in control subjects, and the *USF1* rs3737787T allele was correlated with decreased levels of TC, LDL-C, and TG, as well as a decreased risk of EOCAD in men but not in women. Meanwhile, the rs3737787-alcohol consumption interaction altered serum TC and LDL-C levels in men, serum HDL-C and TC levels in women, and the risk of EOCAD in both men and women. In addition, several haplotypes and interactions of the haplotypes with smoking were associated with different levels of EOCAD risk in men and women. Based on these results, the difference in genotype frequency of the rs3737787 SNP may be an important genetic factor contributing to a significant difference in the susceptibility to EOCAD between men and women.

The allele and genotypic frequencies of the *USF1* rs3737787 SNP in different ethnic/racial groups are not completely clear. By searching the International 1,000 Genomes database (https://www.ncbi.nlm.nih.gov/variation/tools/1000~genomes/), we noticed that the frequencies of the rs3737787T allele and the CT, TT genotypes were 28.57, 44.90, and 6.10% in individuals of Mexican ancestry in Los Angeles, California (MEX); 20.93, 39.53, and 1.16% in Japanese individuals; 12.24, 20.28, and 2.10% in the Maasai in Kinyawa, Kenya (MKK); 26.99, 39.82, and 7.08% in Europeans; 3.10, 6.19, and 0.00% in Sub-Saharan Africans; 6.11, 12.22, and 0.00% in the Luhya in Webuye, Kenya (LWK); 10.20, 20.41, and 0.00% in Americans of African Ancestry in the Southwestern USA (ASW); 23.17, 31.71, and 7.30% in Han Chinese individuals in Beijing (CHB); 12.50, 25.00, and 0.00% in Gujarati Indians in Houston (GIH); and 28.98, 37.50, and 10.23% in the Maasai in Toscans in Italy (TSI). In the present study, the allelic and genotypic frequencies of the *USF1* rs3737787 SNP were significantly different between the control and EOCAD groups. The frequencies of the CT and TT genotypes and T allele in the current study populations were 30.00, 39.40, and 9.90% in controls and 19.90, 31.90, and 4.70% in patients with EOCAD. These findings indicate that the frequency distribution of the *USF1* rs3737787 SNP may display ethnic/racial and population-specific patterns. However, these findings still require further confirmation in other populations or ethnic groups with larger sample sizes.

The correlations between the rs3737787 SNP and serum lipid parameters have been reported in several different ethnic groups. Coon et al. found that *USF1* rs3737787, the most significant genetic variant associated with FCHL, is significantly correlated with serum TG and LDL-C levels in the Utah population (21). Lee et al. found that the *USF1* rs3737787C risk allele is significantly correlated with TG, TC, and ApoB levels, as well as FCHL in Dutch males, and with BMI and serum TG levels in American white males (32). Reiner et al. found that the *USF1* rs3737787T allele correlates with decreased serum TC and LDL-C levels (23). Song et al. found that the *USF1* rs3737787 SNP is associated with TG and Apo E levels in families with T2DM in northern China (49). However, the potential associations between the rs3737787 SNP with serum lipid levels and the risk of EOCAD among the Han population in southern China remain unclear. In the present study, we found that the rs3737787 SNP was significantly correlated with serum LDL-C, TG, and TC levels; male rs3737787T allele carriers maintained lower TC, LDL-C, and TG levels than non-carriers; and the dominant model of the rs3737787 SNP decreased the morbidity of EOCAD in men but not in women. When further exploring the effects of the interactions of SNP–environment factors on serum lipid levels and the risk of EOCAD, we found that the rs3737787-alcohol consumption interaction decreased serum TC and LDL-C levels in men, increased serum HDL-C levels in women, and decreased the risk of EOCAD in both men and women. Thus, the effects of the rs3737787 SNP and the rs3737787-alcohol consumption interaction on serum lipid levels and the risk of EOCAD are specific to ethnicity and sex.

When further exploring the LD among the six SNPs, moderate LD was noticed among the rs3737787, rs2774276, and rs2516839 SNPs. The haplotype analysis revealed that rs3737787C-rs2774276G-rs2516839G is the dominant haplotype, and it correlates with an increased risk of EOCAD in men but not in women. Previous studies suggested that the rs3737787C allele was correlated with an increased risk of early-onset coronary atherosclerosis in young adults (23). According to Laurila et al. the rs2516839A allele is significantly correlated with advanced atherosclerosis of the coronary artery and abdominal aorta (25). Similarly, in the present study, the rs3737787C allele represented a risk allele that was significantly associated with EOCAD in men; however, no significant correlation between the rs2516839 SNP and the risk of EOCAD was observed. Interestingly, the rs3737787C–rs2774276G–rs2516839A haplotype and the interaction between rs3737787C and rs2516839A alleles increased the risk of EOCAD in both men and women. Based on these results, rs2516839A may function as a risk allele synergistically with the rs3737787C allele to increase the risk of EOCAD in individuals carrying both alleles. In addition, we also noticed that the rs3737787T–rs2774276C–rs2516839A haplotype correlated with an increased risk of EOCAD in women. Furthermore, the rs3737787C–rs2774276G–rs2516839A-, rs3737787C–rs2774276G–rs2516839G-, and rs3737787T–rs2774276C–rs2516839A-smoking interactions increased the risk of EOCAD in men and/or women. These results suggested that smoking represents a risk factor that functions synergistically with several risk haplotypes to increase the risk of EOCAD, and the effect of haplotype–environment interactions on the risk of EOCAD is also sex-specific. Thus, in addition to the role of genetic factors, environmental factors such as smoking and the interactions between haplotype and smoking also significantly affect the risk of EOCAD. However, more basic research is still needed to clarify the molecular mechanism underlying the effects of these interactions on the risk of EOCAD.

In recent years, with more in-depth research, researchers have gradually realized that arteriosclerosis is actually a chronic inflammatory process characterized by strong immune activity (41). Previous studies have reported that *USF1*, a transcription factor, not only regulates glucose and lipid metabolism but also regulates immune and stress responses (50) and increases the expression of inflammatory factors by activating the nuclear factor kB (NF-kB) signaling pathway, ultimately leading to the occurrence of inflammatory diseases (51). As shown in the study by Ruuth et al. the inactivation of *USF1* effectively promotes the cholesterol efflux from macrophages and reduces the accumulation of cholesterol in macrophages induced by inflammation to alleviate the progression of atherosclerotic lesions (52). Laurila et al. found that *USF1* knockout effectively ameliorates insulin resistance, dyslipidemia, hepatic steatosis, obesity, and atherosclerosis induced by a high-fat diet in a mouse model (53). Furthermore, Colombo et al. noticed a significant increase in the expression of vascular endothelial growth factor (VEGF) in patients with systemic lupus erythematosus (SLE), and it correlates with accelerated atherosclerosis by functioning as a potent angiogenic and vasoactive molecule (54). Silencing of *USF1* expression increases the expression of VEGF (55). In addition, Li et al. proved that inhibiting the expression of *USF1* significantly reduces the expression of inflammatory factors such as IL-1β, TNF-α, and IL-6, thereby alleviating atherosclerotic inflammatory responses (56). Therefore, *USF1* may be involved in atherosclerosis by regulating the expression of IL-1β, VEGF, TNF-α, and IL-6.

By querying the miRdSNP (57) and SNPinfo (58) databases, we found that the rs3737787 SNP is located in the binding site of miRNAs, including miR-148a-3p, miR-148b-3p, and miR-152-3p, in the 3′UTR of *USF1*. Bu et al. suggested that the rs1056628 SNP in the 3'UTR of the matrix metallopeptidase 9 (MMP9) gene alters the expression levels of the MMP9 mRNA and protein by mediating the binding of miR-491-5p to MMP9 and subsequently affecting the susceptibility of Chinese populations to idiopathic calcium kidney stones (59). Zhou et al. proved that SNPs in the 3′UTR of amyloid precursor protein (APP) alter the regulation of APP expression by miRNAs, including miR-144-3p, miR-101-3p, miR-383-5p, and miR-153-3p, and subsequently modulate the occurrence of Alzheimer's disease (60). However, researchers have not clearly determined whether the rs3737787 SNP alters *USF1* expression levels. In the present study, significantly higher expression of the *USF1* mRNA, TNF-α, IL-1β, and IL-6 was detected in patients with EOCAD than in control subjects; the expression levels of TNF-α, IL-1β, and IL-6 were positively correlated with the *USF1* mRNA expression levels, and rs3737787T carriers maintained lower *USF1* mRNA and IL-1β, TNF-α, and IL-6 expression levels than rs3737787T carriers. These results suggested that the rs3737787 SNP may affect the expression of inflammatory factors such as IL-1β, TNF-α, and IL-6 by mediating the expression of *USF1*, ultimately affecting the risk of EOCAD. The potential underlying molecular mechanism is that the rs3737787T allele enhances the binding of miR-148a-3p, miR-148b-3p, and miR-152-3p to *USF1*, thereby decreasing the expression of *USF1*. However, more *in vivo* and *in vitro* studies are needed to confirm these findings.

The current research may have several limitations. First, compared with some previous genetic studies analyzing large samples, the numbers of controls and patients with EOCAD were relatively small. Second, the vast majority of patients with EOCAD were taking some secondary prevention drugs for CAD that may alter blood lipid levels; thus, a calculation of the correlations between the rs3737787 SNP and serum lipid levels in the EOCAD group is inappropriate. Third, although fasting venous blood collected after a 12 h fast was used to detect serum lipid levels, subjects' long-term dietary habits might affect blood lipid levels and EOCAD risk, and the differences among the subjects' dietary habits were not considered in this study. Finally, the VEGF expression level was not detected in this study, and we have not yet clearly determined whether *USF1* is involved in CAD by regulating VEGF expression.

## Conclusion

The current study revealed that the *USF1* rs3737787 SNP is one of the important genetic factors affecting susceptibility to EOCAD, and the potential mechanism may be that the *USF1* rs3737787T allele alters the binding of miRNAs to the target gene *USF1* to reduce *USF1* mRNA expression, thus affecting blood lipid levels and the expression of inflammatory factors, including IL-1β, TNF-α, and IL-6, and ultimately altering the risk of EOCAD. The *USF1* gene is expected to become a potential therapeutic target for the prevention and treatment of EOCAD.

## Data Availability Statement

The datasets presented in this study can be found in online repositories. The names of the repository/repositories and accession number(s) can be found in the article/Supplementary Material.

## Ethics Statement

The study design was approved by the Ethics Committee of Hunan Provincial People's Hospital (No: LL-20210615-144). The patients/participants provided their written informed consent to participate in this study.

## Author Contributions

P-FZ conceived the study, participated in the design, performed the statistical analyses, and drafted the manuscript. Z-FZ conceived the study, participated in the design, and helped draft the manuscript. H-WP performed the epidemiological survey and collected the samples. L-ZC and PL performed the statistical analyses. PL helped to revise the manuscript. All authors read and approved the final manuscript.

## Funding

This study was supported by the Key Research and Development Program of the Hunan Provincial Science and Technology Department (Grant No. 2019SK2021). The funding agency had no role in the design of the study and collection, analysis, and interpretation of data or in writing the manuscript.

## Conflict of Interest

The authors declare that the research was conducted in the absence of any commercial or financial relationships that could be construed as a potential conflict of interest.

## Publisher's Note

All claims expressed in this article are solely those of the authors and do not necessarily represent those of their affiliated organizations, or those of the publisher, the editors and the reviewers. Any product that may be evaluated in this article, or claim that may be made by its manufacturer, is not guaranteed or endorsed by the publisher.
