# Supplementary material for: Effects of USF1 SNPs and SNP–Environment Interactions on Serum Lipid Profiles and the Risk of Early-Onset Coronary Artery Disease in the Chinese Population
Source: Front Cardiovasc Med. 2022 Jun 15;9:882728. doi: 10.3389/fcvm.2022.882728 (PMC9240353; doi:10.3389/fcvm.2022.882728)
Supplement: Supplementary file 1 [file Data_Sheet_1.pdf]

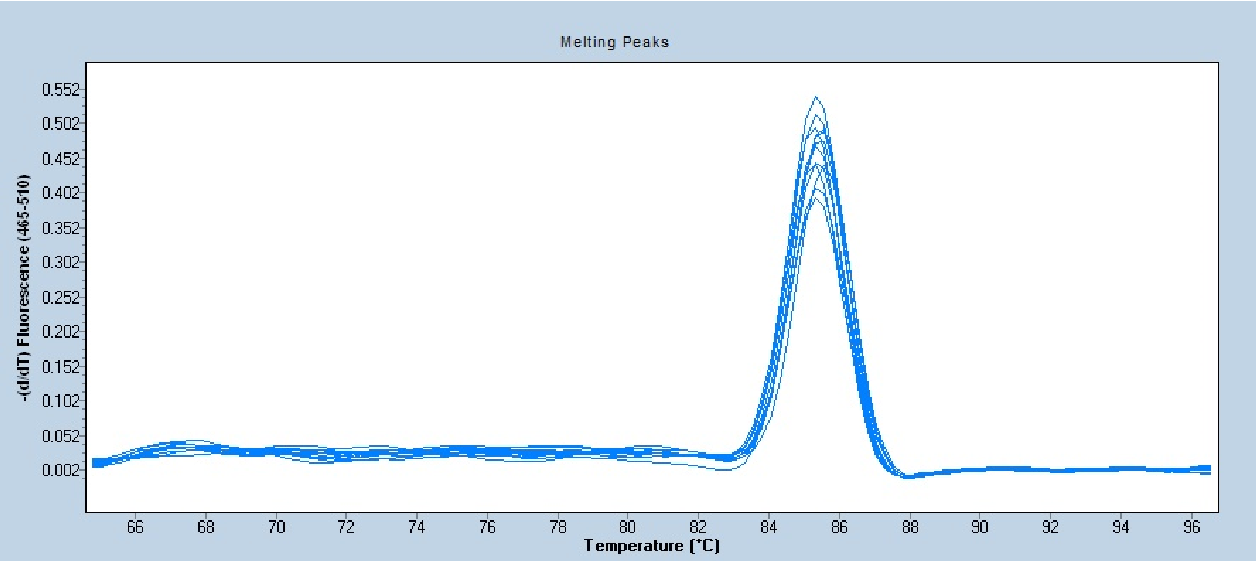

Additional Figure 1: Melting curve analysis of the USF1.

The products of RT-qPCR had a single melting curve indicating the breakdown of only one PCR.

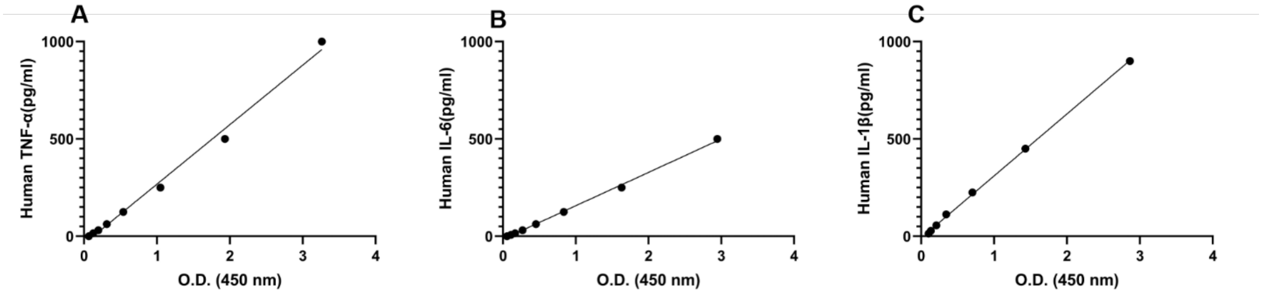

Additional Figure 2: Standard curve of human TNF-α (**A**), IL-6 (**B**) and IL-1β (**C**).

TNF-α, tumor necrosis factor-α; IL-1β, interleukin 1β; IL-6, interleukin 6
